# Australia's National Bowel Cancer Screening Program: does it work for Indigenous Australians?

**DOI:** 10.1186/1471-2458-10-373

**Published:** 2010-06-25

**Authors:** Aliki Christou, Judith M Katzenellenbogen, Sandra C Thompson

**Affiliations:** 1 Centre for International Health, Curtin University of Technology, GPO Box U1987 Perth WA 6845, Australia; 2 Combined Universities Centre for Rural Health, University of Western Australia PO Box 109 Geraldton WA 6531, Australia

## Abstract

**Background:**

Despite a lower incidence of bowel cancer overall, Indigenous Australians are more likely to be diagnosed at an advanced stage when prognosis is poor. Bowel cancer screening is an effective means of reducing incidence and mortality from bowel cancer through early identification and prompt treatment. In 2006, Australia began rolling out a population-based National Bowel Cancer Screening Program (NBCSP) using the Faecal Occult Blood Test. Initial evaluation of the program revealed substantial disparities in bowel cancer screening uptake with Indigenous Australians significantly less likely to participate in screening than the non-Indigenous population.

This paper critically reviews characteristics of the program which may contribute to the discrepancy in screening uptake, and includes an analysis of organisational, structural, and socio-cultural barriers that play a part in the poorer participation of Indigenous and other disadvantaged and minority groups.

**Methods:**

A search was undertaken of peer-reviewed journal articles, government reports, and other grey literature using electronic databases and citation snowballing. Articles were critically evaluated for relevance to themes that addressed the research questions.

**Results:**

The NBCSP is not reaching many Indigenous Australians in the target group, with factors contributing to sub-optimal participation including how participants are selected, the way the screening kit is distributed, the nature of the test and comprehensiveness of its contents, cultural perceptions of cancer and prevailing low levels of knowledge and awareness of bowel cancer and the importance of screening.

**Conclusions:**

Our findings suggest that the population-based approach to implementing bowel cancer screening to the Australian population unintentionally excludes vulnerable minorities, particularly Indigenous and other culturally and linguistically diverse groups. This potentially contributes to exacerbating the already widening disparities in cancer outcomes that exist among Indigenous Australians. Modifications to the program are recommended to facilitate access and participation by Indigenous and other minority populations. Further research is also needed to understand the needs and social and cultural sensitivities of these groups around cancer screening and inform alternative approaches to bowel cancer screening.

## Background

Australia has one of the highest rates of bowel cancer in the world [1]. It is currently the second most commonly diagnosed cancer in men and women, and the third leading cause of cancer-related deaths in the country [2]. While mortality from bowel (also known as colorectal) cancer in the general population has declined in recent years, the situation among Aboriginal and Torres Strait Islanders (hereafter Indigenous) is not so encouraging, with some data showing that bowel cancer is increasing as a significant cause of premature death [3,4]. Similar patterns have been observed in Indigenous populations across the world including Inuit and First Nations in Canada and Maori in New Zealand (N.Z), generally attributed to an increasingly westernised lifestyle [5-7].

Cancer screening aims to identify unrecognized disease in individuals at average risk who are asymptomatic. Only 40% of bowel cancers are detected in their early stages primarily because very few if any symptoms manifest until the cancer reaches an advanced stage, thus making screening an invaluable tool for cancer prevention [2]. Early diagnosis and intervention can substantially improve treatment outcomes and survival in patients diagnosed with bowel cancer.

Commencing with Phase 1 in 2006, Australia implemented a large, nation-wide organized screening program for the prevention and early detection of bowel cancer. Population screening programs require high uptake by the target group in order to make an impact on health and benefit the population being screened [8], however, participation by Indigenous Australians in the program has been poor [9,10]. By recognising and addressing barriers to screening uptake, significant changes were made to the national cervical cancer screening program to accommodate for Indigenous women's needs, and we argue that a similar approach is necessary for the National Bowel Cancer Screening Program (NBCSP) [11].

This review provides a descriptive and critical analysis of Australia's NBCSP in order to identify key characteristics of the program that could inadvertently exclude and impact on participation by Indigenous people and other ethnic minorities. Specifically, it examines organizational aspects and socio-cultural barriers facing Indigenous Australians in accessing and engaging in a program which does not take into account their social and cultural diversity, and healthcare access inequities.

While our focus is on characteristics of the program that may discourage and hinder participation by Indigenous Australian, the issues highlighted are also relevant to other ethnic minority and disadvantaged groups and people living in remote locations. Such issues need open discussion in order to catalyse policy and program changes to ensure a more equitable and accessible program for all Australians.

## Methods and Results

### Search strategy and approach

This review draws upon the existing literature to identify problematic areas in the Australian NBCSP that need to be addressed to optimize the program for Indigenous people. The dearth of information available on Indigenous populations in relation to bowel cancer screening particularly in Australia, required that we broaden our examination to studies with minority and ethnic groups and also take lessons from breast and cervical screening studies which have been more extensively studied in Indigenous populations.

We undertook a search of the literature for material relevant to the following research questions:

• What is the current state of bowel cancer and bowel cancer screening among Indigenous Australians?

• How is the NBCSP disseminated and what may be influencing Indigenous participation?

• Are there any aspects of the program which appear problematic and which could be modified to improve participation?

Similar to the critical interpretive synthesis approach, the process of literature searching was dynamic and reflexive with themes emerging as the literature was searched. This served to shape the content of the review [12]. In contrast with systematic reviews, where inclusion and exclusion criteria are defined at the outset, our approach was more appropriate in the context of limited availability of literature. Yet, our critique drew upon elements of a systematic review, including an initial methodical and structured search of the literature using electronic databases to synthesise and appraise the information available.

Peer reviewed journal articles were selected following a search of electronic databases including Proquest, Science Direct, Google Scholar, PubMed, Medline, Informit and ISI Web of Knowledge. Key search terms used included a combination of the following: Indigenous or Aboriginal with cancer screening, bowel cancer, colorectal cancer, colon cancer with screening; ethnic or racial minorities and disparities in colorectal or bowel cancer screening. Other search terms included, Native American and Alaska Native, Maori, First Nations, Inuit and Metis, ethnicity or ethnic minorities with colorectal or bowel cancer screening. Additional articles were obtained through citation snowballing. The Australian Indigenous Health *InfoNet*, the Australian Institute of Health and Welfare, the Australian Bureau of Statistics, Australian and State Cancer Council websites and published and unpublished government reports also provided useful information and links.

Publications were considered for inclusion if they addressed at least one of the above research questions and contained information about colorectal cancer incidence, mortality and survival or colorectal cancer screening participation in Indigenous populations and racial/ethnic minority groups. Articles examining knowledge, attitudes, beliefs and barriers toward colorectal cancer screening uptake and interventions to improve screening uptake in these populations were also included. Literature was not limited to Indigenous Australians or Indigenous populations because of the dearth of studies available. As literature on colorectal cancer screening in Indigenous Australians was minimal, papers looking at breast and cervical screening practices were included to identify participation levels, barriers and interventions for increasing uptake. Papers that did not refer to an Indigenous, minority or disadvantaged group were excluded.

### 1. Descriptive review

#### 1.1 Bowel cancer in Indigenous Australians- epidemiology and data quality

Although bowel cancer is rarely noted as a significant health issues for Indigenous Australians, it is the third most common cancer after lung and breast in women, and lung and prostate in men, accounting for about 10% and 9% of all cancers respectively [13]. However, the lack of national data has meant that little information is available surrounding the impact of bowel cancer on Indigenous Australians. The Australian Institute of Health and Welfare (AIHW) reports that incidence and deaths from bowel cancer are much lower than the non-Indigenous population [13], although the true magnitude of bowel cancer is not clear due to problems associated with under-ascertainment of Indigenous status in cancer data collection registries. The Australian Government recognizes three criteria as determining Aboriginal and Torres Strait Islander status: - Indigenous descent; self-identification as an Indigenous person and acceptance of the individual as Indigenous by the Indigenous community [14]. Administrative data collections generally focus on self-identification to classify an individual as Indigenous, and while ascertainment of Indigenous status is improving over time in Australia, under ascertainment is an acknowledged problem with gaps in the data that affect the accuracy of statistics recorded [14]. Consequently, Indigenous cancers are thought to be underestimated by up to 20% and mortality up to 35% [13,15,16].

The observed low incidence and mortality from bowel cancer may be influenced by a number of other factors, including the high rate of incomplete or inadequate death certification and the large proportion of cancers of undefined primary site in registered Indigenous cancer records [13,17]. Additionally, as cancer risk increases with advancing age, the substantially lower life expectancy of Indigenous Australians means they may die from other chronic conditions prior to cancer diagnosis [18]. Lower participation in cancer screening and the possibility that Indigenous people with bowel cancer may avoid seeking medical care for symptoms or accessing treatment could also reduce ascertainment of disease [19-22].

Age-standardized incidence rates from 2002-2004 suggest that the rate of bowel cancer in Indigenous Australians is about half that of the non-Indigenous population (39.7 vs 76.4 per 100 000 in males and 36.6 vs 52.4 in females; rate ratios are 0.5 for males and 0.7 for females) [13]. Incidence data collected from cancer registries (2002-2006) in three Australian states which have the most reliable data collections gave a slightly lower overall age standardized rate of 29.9 per 100 000 among Indigenous people compared to 64.7 per 100 000 in non-Indigenous [16]. However, these figures mask higher rates that exist in younger Indigenous Australians. Age-specific rates show that incidence was similar in non-Indigenous and Indigenous people up until age 50. Notably, men in the 40-49 year age group were found to have higher rates of rectal cancer than non-Indigenous men, attributed to greater consumption of alcohol. Supporting the increased burden at a younger age are findings from the Northern Territory (NT) indicating that incidence rate ratios were higher in Indigenous Australians under 64 years compared to those over 64 years (0.6 vs 0.2; 1991-2001) [3,4].

Colorectal cancer (CRC) mortality estimates vary in different jurisdictions. In a NT study (1987-95), age standardised mortality rates in Indigenous people were about half that of non-Indigenous people [22], while in rural and remote Queensland and in New South Wales mortality rates were not significantly different between Indigenous and non-Indigenous people [15,23]. A more recent NT analysis showed mortality rates were similar in Indigenous compared to non-Indigenous people under 65 years, but substantially lower in older ages, suggesting an interaction between age and Indigenous status [24]. This was further supported by recent results from four Australian states, where despite somewhat lower age standardised death rates in Indigenous people (17.9 per 100 000 compared to 19.8 in the non-Indigenous population) age-specific mortality rates were actually higher in Indigenous people under 60 years compared to the non-Indigenous population [25].

Indigenous Australians generally have higher mortality following a cancer diagnosis compared to non-Indigenous Australians, especially for those cancers which are amenable to early diagnosis and treatment [22,26]. Later staging at diagnosis means patients present with more advanced disease and experience poorer treatment outcomes; Indigenous people also appear less likely to undergo and comply with treatment [19,21]. This has also been shown to be true for CRC [21]. Thus, once diagnosed, Indigenous people are more likely to die from CRC compared to their non-Indigenous counterparts as reflected in the poorer five year survival rate. Condon et al. [21] found that nearly 90% of Indigenous people with CRC in the NT presented with advanced disease at diagnosis as opposed to 68% of the non-Indigenous group, and that five year survival was only 31% compared to 55% in the non-Indigenous group [4]. This pattern is consistent with CRC mortality and survival observed in Maori, Native Americans, Native Hawaiians, First Nations and Inuit [6,27-31].

Reasons behind late presentation and delayed diagnosis have been explored in studies examining breast and cervical cancer in Indigenous people and include a low awareness of symptoms, delay in seeking medical advice, reduced access to and low quality primary care, diagnostic and specialist services, and a disinclination to seek advice for symptoms because of certain beliefs regarding cancer and the chance of successful treatment [4,32]. Late presentation and poor survival is also influenced by communication and cultural barriers which may impact the effectiveness and choice of treatment, as well as the decision to engage in preventive and diagnostic activities including cancer screening [19,20,33]. The presence of other chronic diseases or environmental conditions also limits provision of treatment due to the risk of complications [4,21,26,34].

National cancer screening programs can partly address the survival discrepancy in Indigenous Australians by facilitating early diagnosis. If implemented in a culturally sensitive manner, cancer screening can aid in reducing mortality from the disease [26]. This has been demonstrated in the NT and WA where cervical cancer mortality declined substantially following the provision of a culturally appropriate holistic cancer screening services [35-37]. However, Australia's national bowel cancer screening program in its current form has not taken such an approach, reflected in the very low uptake by the Indigenous population.

#### 1.2 Bowel cancer screening and Australia's National Bowel Cancer Screening Program

Bowel cancer screening is an effective means of reducing the mortality and burden of CRC in the community through the early detection of abnormal changes in the bowel [38,39]. It is an ideal malignancy to target for population screening and fits the World Health Organisation's criteria for suitability for population based screening [38]. Importantly, it has an identifiable early precursor lesion, usually in the form of an adenomatous polyp which in most cases is asymptomatic; it has a long latency period (for a polyp to become cancerous), thus providing an opportunity for early diagnosis; and there is a high cure rate if found with available effective treatment. Five year survival rates can be almost 90% if a tumour is detected in the earliest stages (Stage A) while still asymptomatic and localized. Once cancer has proceeded through the bowel wall, to the lymph nodes and beyond, five year survival is reduced to only 7% [38,40]. In South Australia (SA), a monitoring system showed that only 15% of bowel cancers were detected at Stage A indicating that substantial potential improvements in survival could be achieved with earlier diagnosis [41].

Australia is one of a few countries implementing a formal, government funded, population-based CRC screening program - others include the UK, Canada, France, Italy and Finland [42-44]. Following a bowel cancer screening pilot program in 2002-2004 which examined the feasibility, acceptability and cost-effectiveness of the program in the Australian community, the first phase of the National program was rolled out in 2006 and was offered to people turning 55 and 65 years of age. The second phase began in July 2008 and expanded eligibility to all men and women turning 50, 55 or 65. The program targets only specific ages due to limited funding and to ensure that health services can cope with the increased service demands [9]. The target population age range was chosen based on the National Health and Medical Research Council guidelines which indicated the risk of developing bowel cancer increases significantly over the age of 50 years, with over 90% of those diagnosed with CRC in Australia aged over 50 [25].

Funding for the NBCSP has been secured only until 2011, allowing for once-off testing of the population - despite the fact that the mortality benefit associated with screening depends on regular participation [45]. The program is freely available to people aged 50, 55 and 65 years only and no plans have been announced for second yearly screening. If fully implemented to a broader age range and with ongoing screening rounds, the program could potentially save up to 2,000 lives per year in Australia [46]. Furthermore, sequential screening can also provide an estimation of incidence (new disease) since last screen, as opposed to the initial screening round which cannot differentiate between new and old cases.

The Bowel Cancer Screening Implementation Committee chose the faecal occult blood test (FOBT), a simple non-invasive test that detects small amounts of blood in the bowel motion, as the test of choice for population screening in Australia. The presence of blood in faeces is a sign that abnormalities such as a precancerous lesion or cancer may be present in the bowel. Landmark trials have shown that regular population screening using FOBT can reduce the risk of death from CRC by up to 33% in those who (regularly) participate [39,45,47]. FOBT is the only screening test for CRC where evidence from RCTs demonstrated a reduction in mortality [48], although the trials used the guaiac FOBT (gFOBT) as opposed to immunochemical FOBTs (iFOBT), the test used in Australia. One recently completed RCT using the iFOBT demonstrated increased detection and compliance, although long-term mortality benefits have yet to be demonstrated [49].

**Figure 1:**
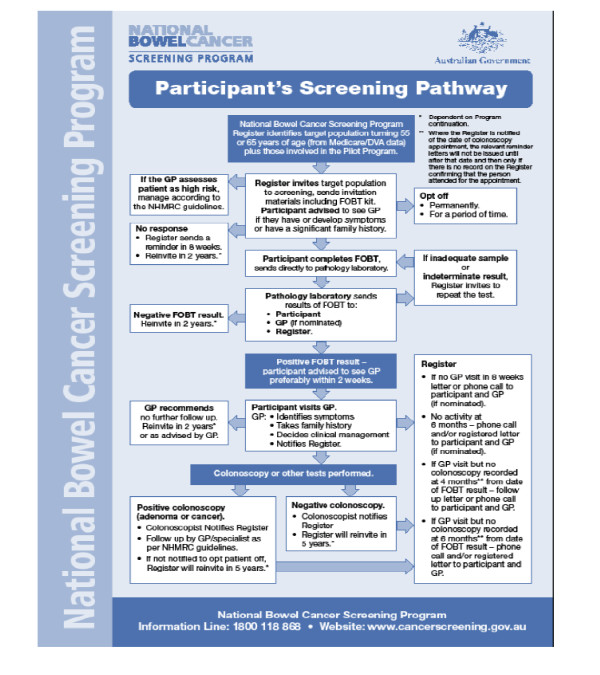
**Participants screening pathway in the National Bowel Cancer Screening Program (taken from **[10]**)**. See separate file

The screening pathway is shown in Figure 1. Screening test kits are mailed to individuals following identification of eligible program participants from enrolment in the national healthcare insurance scheme (Medicare). Kits are completed at home by taking samples of two consecutive bowel motions and mailed back to a pathology laboratory for testing without cost to the patient. Results are sent back to participants and their nominated GP (if one has been nominated). For positive tests, a letter advises participants to visit their GP to discuss a follow-up procedure, namely colonoscopy. A bowel cancer screening register has been established within Medicare to assist in follow-up of those with positive tests not undergoing colonoscopies, rescreening of participants, and to aid data collection, monitoring and evaluation [9]. The data obtained are collected via forms filled in by the individual, their GP, colonoscopist and others involved. As completion of the forms is not compulsory, a large amount of information is incomplete or missing [25].

The sensitivity and specificity of FOBT varies by test type and manufacturer. Sensitivity is in the order of 60-85% for iFOBTs when used regularly within a program, although with once-off testing this drops to 30-50% [48]. The iFOBT (MagStream 1000/Hem SP) currently used in the Australian program has a sensitivity of 66% for cancer and 20% for polyps over 1 cm [50-52]. Hence, many false positives occur, resulting in high numbers of follow-up colonoscopies. Of those who have a positive FOBT, about 5-10% will have CRC, and 20-35% will have an adenoma identified [41]. Test outcomes and the ability of the test to identify CRC are not usually fully understood or made apparent to the public, yet are important when weighing up the risks and benefits associated with participation in the program [53].

In 2009, nearly half a million testing kits were recalled and the program was temporarily suspended for six months after an unusual decline in the number of positive test results, assumed to be due to faulty kits. Testing resumed in November 2009 with a new kit that was initially distributed to re-screen those who received the faulty kits [54]. However, the new kit is recommended for use only up to 30 degrees Celsius creating potential problems for many regions, particularly in the north of Australia where a large proportion of Indigenous people reside. The new screening kit will not be sent to these regions initially, creating even further inequities in access. Much greater thought and effort needs to be put into the problem of differential access to the program.

#### 1.3 Indigenous participation and test profile - results of National Bowel Cancer Screening Program monitoring reports, 2006-2008

Adequate levels of participation are essential for the success of a screening program in reducing mortality from CRC and for population benefit equating to that attained in randomized controlled trials [8]. It is also an important contributor to the cost-effectiveness of the program in terms of number of lives saved and costs saved by averting a cancer diagnosis [8,55]. Monitoring reports completed in 2007, 2008 and 2009 details performance of the program from 2006-2008 [10,25,56].

Overall participation in the Australian NBCSP was 37.6% by the end of 2008 however, participation was lower than that observed during the pilot (45.4%). The average positivity rate was 6.6%, significantly higher among men than women which is reflected in higher incidence rates [25]. Table 1 details variation in participation, positivity rate and proportion of correctly completed tests according to demographic factors.

**Table 1:** Participation in the NBCSP according to Indigenous status, gender, SES, geographic location and language spoken

Participation rate							
*Gender*	**Males**	**Females**	**Total**				Females 1.2 times more likely to participate than males
	36.2 *(2008)	43.3* (2008)	39.7				
	36.0 (2009)	42.6 (2009)	39.3				
							
*Indigenous Status#*	**Non-Indigenous**	**Indigenous**	**Total**				Indigenous people are 2.3 times less likely to participate Than non-Indigenous people
	38.6 (2008)*	17*(2008)	38.3				
	37.0*	17.0*					
	45.4 (Pilot)						
*SES*	**Lowest SES**	**Highest SES**					Participation significantly lower in the most disadvantaged quintile compared to any other quintile
	37.5*(2008)	41* (2008)					
							
*Geographic Location*	**Very Remote**	**Remote**	**Outer Regional**	**Inner Regional**	**Cities**	**Total**	Proportion of those who participate was significantly lower in remote and very remote compared to the national level. Participation was significantly higher in inner regional compared to other areas.
	25.6*(2008)	35.5	40.9	43.7	38.4	39.7*	
	25.0(2009)		39.1	40.1			
							
*Spoken language*	**English as preferred language**	**Other language preferred**					Those whose preferred correspondence language was English were 1.6 times (2008) and 2.9 times (2009) more likely to participate
	42.2* (2008)	27.0* (2008)					
	41.1* (2009)	14.0* (2009)					

**FOBT Positivity rate**							

*Gender*	**Males**	**Females**	**Total**				
	8.9*	6.4*	7.5 (2008)				
	7.7	5.7	6.6 (2009)				
							
*Indigenous Status#*	**Non-Indigenous**	**Indigenous**					Not statistically significant difference due to small numbers
	7.5 (2008)	8.6 (2008)					
	6.6 (2009)	8.1 (2009)					
							
*Geographic location*	**Very Remote**	**Remote**	**Outer Regional**	**Inner Regional**	**Cities**	**Total**	
	8.7*(2008)	8.7*(2008)	8.6*	7.9*	7.2*	7.5	
	8.4 (2009)	7.8 (2009)	7.3 (2009)		6.4 (2009)		
*SES*	**Low SES**	**Highest SES**					
	7.8 (2009)	5.5 (2009)					

Proportion of correctly completed tests							

*Indigenous status#*	**Non-Indigenous**	**Indigenous**					Significantly lower in Indigenous compared to non-Indigenous participants. Indigenous females aged over 65 have lowest correctly completed tests
	96.3 *	93.9 *					
							
*Preferrred language*	**English**	**Other**	**Total**				Significantly lower number of correctly completed tests among those whose preferred language is not English
	96.7*	91.7 *	96.2				
							
*Disability status/activity limitation#*	**Severe or profound**	**None**	**Total**				Significantly lower number of correctly completed tests in those with activity limitation
	90.6*	96.5*	96.2				
							
*Geographic location*	**Very Remote**	**Remote**	**Outer regional**	**Inner regional**	**Major cities**	**Total**	
	96.0	97.4*	96.8*	96.9*	95.8*	96.2	

**Primary Practitioner visits after a positive test**							

							
*Indigenous status#*	**Indigenous**	**Non-Indigenous**	**Total**				No significant difference
	46.4	43.7	43.2				
							
*Preferred language*	**English**	**Other language**					Those with a preferred language other than English were significantly less likely to visit a GP after a positive test result.
	43.6*	40.0*					
*SES*	**Lowest SES**	**Highest SES**					Those in the most disadvantaged quintile were significantly more likely to see a GP following a positive test result
	43.9*	40.4*					

Percentages are the number of people participating as a proportion of the total number of the eligible population who were sent invitation to screen

2008 - 2008 Monitoring report reports performance of NBCSP from 30 June 2006-7 August 2008. Included 55 and 65 year olds

2009 - 2009 Monitoring report reports performance of NBCSP from January 2008- 31 December 2008. Included 50, 55 and 65 year olds

*significant difference exists

#Disability status and Indigenous status were self reported measures

Analyses up until mid-2008 showed Indigenous participation was 17% - less than half the non-Indigenous rate [10]. In the 2009 monitoring report which documented results from 2008 only, Indigenous people were 2.2 times less likely than non-Indigenous to participate in the screening program [25]. However, Indigenous identification of participants in the program was through self-report and was completed by only 63% of participants. Another important finding from the 2008 report was that Indigenous people were significantly less likely to complete the FOBT correctly, with Indigenous females aged over 65 years having the lowest rate of correctly completed tests [10]. Indigenous people also had higher FOBT positivity rates although this was not significant due to the small sample size [25]. The proportion of correctly completed tests was also significantly lower for people who nominated a language other than English as their preferred language [10]. Significant differences were also observed according to geographic location, with inner regional areas having the highest rates of participation while those in remote and very remote areas (where 26% of Indigenous Australians reside) were significantly less likely to participate. Participation also fell with lower SES [10], and those in the most disadvantaged lowest quintile of the population were significantly less likely to follow up a positive result with their GP. Indigenous Australians are disproportionately represented in this quintile.

Substantial numbers of unreturned health provider forms make it impossible to determine how many Indigenous (or other) people with positive FOBTs went on to complete a colonoscopy. For the entire program, only 42% of participants with a positive FOBT had a colonoscopy recorded. The remaining participants either did not have a colonoscopy or the colonoscopy report was not returned [25]. Lack of quality surveillance information compromises the assessment of actual population benefits and the impact on the incidence and mortality from CRC, particularly on population subgroups. Despite this, a recent analysis by Ananda et al [57] assessing the initial impact of the NBCSP from mid 2006 to mid-2008, reported its success in identifying CRC at an earlier stage. This study utilised linked data from hospitals across Australia to gather information on CRC cases reported by surgeons and found that 40% of cancers identified through the NBCSP (N = 40) were at Stage 1- the most treatable stage, compared to 14% among individuals who presented with symptoms to their doctor [57].

### Critical Review and Interpretation

#### 1.1 Factors contributing to poor uptake of bowel cancer screening in Indigenous populations

An individual's decision to engage in CRC screening is complex and influenced by socio-cultural, historical, system and organizational factors, and individual behavioural factors - all of which impact on screening uptake at a population level [8,58]. Unfortunately, the design of screening programs often does not reflect the diversity of social and cultural values of a nation's population, creating significant barriers to participation in CRC screening.

Bowel cancer screening using the FOBT differs quite considerably from other screening programs in its complexity, and is unique in many ways which makes it a difficult program to promote. A self-administered test requires that one complete it at home and return it, hence relying on individuals to take initiative and follow instructions. Moreover, people may find it an unpleasant test to complete, and the first faecal sample must be stored appropriately until the other is taken. CRC screening targets both males and females, which not only increases the size of the target population, but men's lack of previous involvement in screening programs poses additional challenges for recruitment. There is also some confusion surrounding the age groups for screening with inadequate understanding by and explanation to participants as to why only certain people are chosen. As with most screening tests the FOBT is not diagnostic and only identifies those at high risk, so further investigation is necessary for a definitive diagnosis to be made, making the process longer and more stressful for the client. Thus, participation must be addressed along the entire screening pathway - at the screening stage, if further investigation with colonoscopy is required, then again when treatment is required. Where ongoing screening is incorporated into the program, difficulties arise getting people to return for subsequent two-yearly screens.

Lower screening uptake by certain racial/ethnic and minority groups has consistently been documented in countries implementing CRC screening programs [59-62]. Lower participation also occurs among the less educated, lower income groups, and those from non-English speaking backgrounds [10,58,63,64]. Studies controlling for SES and other factors such as distance from services and education show that race and ethnicity are independent predictors of CRC screening uptake [28,65].

Research reveals that beliefs and attitudes of ethnic and minority groups in regard to cancer and cancer screening can differ quite significantly from the majority mainstream population which may have important implications for participation in cancer prevention efforts and treatment [17]. A number of studies in the USA have identified differing views and beliefs that could act as motivators or barriers to CRC screening [66-68]. A study of American Indian and Alaska Natives (AI/AN) found that having a family history of cancer, higher education and income, presence of other chronic conditions, speaking English at home, urban residence, and older age were predictors of CRC screening participation [69]. Researchers examining other ethnic groups have identified that being physically active, having a greater perceived susceptibility to CRC, a doctor's recommendation and participation in other screening programs as important predictors of screening adherence [70], while lack of knowledge, lower perceived risk and perceived self-efficacy, inaccurate beliefs, language and communication barriers, fatalism, the belief that screening is not necessary without symptoms, lower levels of utilisation of health services, the failure of a doctor to recommend screening and cost are significant barriers to screening [66,67,71,72].

Many other developed countries with Indigenous populations are in the beginning stages of CRC screening and while uptake by Indigenous populations has not been extensively studied, early data also indicate lower participation. Table 2 shows differences in CRC screening participation by Indigenous compared to the non-Indigenous population in four industrialised countries, outlining variations in screening recommendations, the type of screening test and how the screening is targeting individuals.

**Table 2:** Differences between CRC and CRC screening programs in selected industrialised countries with disadvantaged Indigenous populations

Country	Organised Screening Program	CRC Incidence	CRC Mortality	Screening recommendations/characteristics	Participation by Indigenous groups
		(per 100 000)	(per 100 000)		
		Indigenous vs non-Indigenous population	Indigenous vs non-Indigenous population		
Australia	Yes	36.6 vs 52.4 [13] for females**	17.9 vs 19.8^^[115]	Free once-off iFOBT For those aged 50, 55 & 65 yrs	17% compared to 39% in non-Indigenous [115,10]
				Kit posted to all in these age groups	
		39.9 vs 76.4 [13] for males**		Currently only a once-off test	

New Zealand	No	15.5 vs 24.1*[116]	8.8 vs 9.8*[116]	No program in place yet	No data available
		Once diagnosed with CRC, Maori are two thirds more likely to die	Relative risk of mortality in Maori is 1.24 after adjusting for age, sex and stage [6]	Opportunistic screening only	
		Mortality: Incidence ratio:			
57% Maori vs 41% in non-Maori					

Canada	Yes	37.2 vs 34.8 for females^c^	16.1 vs 18.4 [118]^b^	FOBT (guaiac) every 2 years	30% adherence in general population but no specific data on Indigenous participation [94]
	(in certain provinces only ie. Ontario and Alberta)	55.1 vs 67 for males^c ^[117]		For those aged 50-74 yrs.	
				Kit obtained free from health care provider.	
				Information sheet available in Inuit language.	

USA	No	33.9 (AI/AN ranged from 17.1-106.2) vs 53.2 (non Hispanic whites) [119,120]^	17.9 (AI/AN) vs 21.0 (USA all races)^a ^[121]	Recommendation by professional organisation for either;	Any CRC screening in last 2 years:
	(Recommendations in place but no organised or national program. Primarily opportunistic and some state programmes exist)		Mortality rate ratio: 1.15 vs 0.89 (10)^a^	Annual FOBT or	38.1% AI/AN vs 58.5% non-Hispanic white [122]
				5 yearly flexible sigmoidoscopy or	FOBT: 5.8% (AI/AN) vs 12.6% (white) [120]
				combination of the above two, or	Sigmoidoscopy or colonoscopy:
				10 yearly colonoscopy	31.7 (AI/AN) vs 45.8 (white) [120]
				For everyone over 50 yrs [48]	Endoscopy or FOBT: 34.4 vs 49.5 [120]

All figures are per 100 000 and are age-standardised

Abbreviations

AI/AN- American Indian/Alaska Native

iFOBT- immunochemical FOBT

gFOBT- guiac FOBT

^ 1999-2004 Included only those that attended Indian Health Services and limited to Contract Health Service Delivery Area Counties. Standardised to 2000 USA population

^^ Age standardized mortality rate from 2002-2006 Queensland, Western Australia, South Australia and Northern Territory combined

** Age standardized incidence rate 2000-2004. Data for NSW, Vic., Qld, WA, SA and NT combined

* 1996-2001 Standardised to Maori population

^a ^Rate adjusted to the 2000 USA standard population

^b ^Age standardized to the 1991 Canadian Population. Mortality rates are from 2000 for First Nations and 2001 for Canada. First Nation rates are for on reserve but include the off reserve population for British Columbia and Alberta

^c^Age standardized to the 1991 Canadian population. Rates are from 1997-2001

Cancer screening programs have had variable success among Indigenous people in Australia. The low participation rates observed for the NBCSP reflect similarly poor engagement of Indigenous women in cervical and breast screening programs, with participation in the order of 30-35% compared to 55-60% in non-Indigenous women [3,37,73]. Indigenous communities face several barriers to accessing cancer-related health services, including early detection programs [74,75]. Evidence from studies assessing breast and cervical screening uptake by Indigenous women in Australia suggest that participation is influenced by multiple structural, socio-cultural and behavioural factors similar to those described above (see Table 3), including poor knowledge and awareness of cancer and the benefits of screening prevention services, culturally insensitive services, shame and fear regarding cancer, lack of Indigenous or female service providers, distrust of the mainstream providers, low literacy, poor coordination of services as well as distance and access barriers [3,17,76-78]. The absence of sufficient Indigenous-specific, culturally relevant, educational resources has also been identified as an important factor impeding cancer prevention efforts [74,79-81]. To date, no published studies in Australia explore barriers specifically towards bowel cancer screening in Indigenous people other than an evaluation conducted following the pilot program in 2004 which interviewed only 15 people - 8 participants and 7 non-participants [82].

**Table 3:** Barriers to cancer screening uptake as well as follow-up and treatment in Indigenous populations

Socio-cultural and behavioural barriers	Structural barriers
**Individual barriers**	**Access barriers**
	
Poor knowledge and awareness of cancer and screening services	Poor coordination of services, from screening to follow-up and treatment
Low levels of health literacy	Lack of transportation
Language/literacy barriers	Distance barriers/rural residence
Low perceived risk	Frequent moving, changing address
Negative attitude	Child care commitments (family responsibilities)
Worry or fear of cancer	Inflexible clinic schedules
Fatalism regarding cancer	Lack of Indigenous staff
Low priority of screening	Difficulties negotiating/communicating with providers and organizations due to language/literacy or cultural differences
Perceived self efficacy	
Lack of appropriate health information	
Presence of co-morbidities	Lack of health promotion material in Indigenous languages
History of racism and distrust in medical institutions	
Discomfort with mainstream services/alienating hospital environment	
Absence of holistic, culturally appropriate cancer services	
	**Economic**
	Cost of seeing a GP including transport
	Unsure of potential costs of follow-up and treatment
	Costs for travel and accommodation to hospital
	
	**Provider-related**
	
	Lack of understanding of cultural needs
	Poor identification of Indigenous status
	Lack of appropriate resources

However, a study of Italian migrants in Australia found similar issues and beliefs to Indigenous Australians - fatalism, English proficiency, fear of cancer and finding out they have cancer, inadequate knowledge and misconceptions of causes of bowel cancer [83]. Weber [84] also explored uptake of cancer screening in Australian migrants and found a significantly lower participation rate in bowel cancer screening among Asian men, although as the authors noted, the NBCSP had not begun in Australia at the time of the survey [84]. This highlights the importance of identifying the needs and preferences of minority groups so as to appropriately target them for bowel cancer screening. To successfully target messages, educational resources and interventions for increasing uptake requires understanding of the current knowledge, attitudes and beliefs of the community of interest.

Potentially, screening could exacerbate inequalities in health as a result of less participation by those of lower SES or ethnic minority groups, including Indigenous populations [8]. A recently completed study in S.A has shown disparities in participation in the NBCSP according to SES, gender and age [85]. Although many countries are examining ways of increasing participation by disadvantaged groups, only N.Z has taken a serious interest in avoiding increasing health inequalities before implementing any sort of CRC screening program that may negatively impact on the health of the Maori population [86]. A CRC screening advisory group in N.Z recommended that any screening program if implemented must: adopt a holistic approach to health and wellbeing; establish partnerships with relevant Maori health organisations; incorporate Maori language in information and health promotion materials; and ensure participation of Maori in the planning and dissemination of the program [87]. Such an inclusive approach with consultation and involvement of the community is likely to have a positive impact on participation and should be explored further in Australia.

#### 2.2 Findings from the evaluation of the bowel cancer screening pilot program

A qualitative evaluation undertaken in 2005 of opinions, attitudes and behaviours influencing participation or non-participation in the pilot bowel cancer screening program is the only available review which provides some insight into Indigenous people's view of the bowel cancer screening program, its associated screening kit, and reasons behind their decision to participate or not [82]. However, only a very small number of Indigenous people were involved (8 participants and 7 non-participants) and the target population of Indigenous people was not considered representative, so the findings need to be interpreted with caution.

The main issues that emerged were that lack of awareness and knowledge of bowel cancer and related screening options as key barriers to participation. There was a lack of understanding of bowel cancer and the benefits of screening and early detection, with many unable to distinguish bowel cancer from other cancers. Not having any symptoms and no family history of cancer were reasons given for non-participation. In addition, there were doubts about the efficacy of the test and some had concerns about physically collecting the stool samples. Rejection of the kit was often the result of anxiety and confusion. The analysis showed those who completed the kit required much encouragement and support from health workers and GPs as they believed that they did not have the ability to correctly complete the test. Indigenous participants did not discuss the test with family members nor did they actively seek assistance from health professionals [82]. Indigenous people that participated were perceived to be more proactive, in control of their health, and more literate. Participation was influenced by personal experiences such as knowing someone with CRC, degree of community engagement and access to information in a known language. Participants in the pilot tended to have a better understanding of why they were receiving the kit and the benefits of the screening as they were more likely to have seen promotions and media campaigns prior to receiving the kit and so had an idea of what to expect and were not surprised when they received it. This was an important trigger for participation, as was contact and encouragement from community health workers, the fact the test was free, and a sense of privilege felt for being chosen for participation. However, this latter factor also instigated fear and anxiety in some people who felt that they were at risk because they were targeted individually [82].

Additional barriers identified were similar to other screening programs and included a fear of cancer and the belief that cancer essentially means death. This finding is consistent with research conducted into the perceptions of cancer among Indigenous people [88]. There is often a social stigma attached to cancer among Indigenous people [17] which may prevent people from utilizing screening.

Bowel cancer screening has not had a strong advertising campaign to increase public education and awareness, so that many people receive the test without being aware of its purpose. Poor knowledge and understanding of the program was highlighted as a major barrier to participation in the pilot [82]. A study [89] assessing television coverage of CRC concluded that the disease is under-reported in the media relative to other cancers, despite incidence and mortality rates being high. The absence of celebrities speaking publicly of their CRC experience was also noted, with publicity of affected celebrities highly effective in influencing the public's perception and increasing uptake of screening [90]. The 2005 evaluation report echoed this need for more extensive media coverage as a means to target hard-to-reach populations [82]. Unfortunately, the program has attracted some negative publicity since the incident with faulty kits which has not helped the case for promoting CRC screening.

#### 2.3 How does the NBCSP exclude Aboriginal and Torres Strait Islanders?

Several features in the way the NBCSP operates, including downstream activities of follow-up and treatment, make access for Indigenous people particularly challenging. Many of these issues were highlighted in the 2005 evaluation report [91], yet to date no changes have been made to the program. Outlined below and summarised in Table 4 are specific elements of the program related to the way it is organised and disseminated that could contribute to unintentionally discouraging participation by Indigenous Australians. Particular socio-cultural barriers are also explored in relation to how they impact on program participation.

**Table 4:** Organisational and structural characteristics of the NBCSP that may exclude participation by Indigenous and disadvantaged populations

1. Medicare enrolment requirement
2. Postal route of FOBT screening kit distribution
3. Role of the General Practitioner
4. Target age group
5. Health information systems around recording Indigenous status
6. Other logistical issues- privacy, storage and test viability
7. Literacy requirement
8. Nature of the screening test
9. Barriers to compliance with follow-up and treatment

##### i) Medicare enrolment requirement

Enrolment with Australia's universal health insurance program, Medicare, determines who receives a screening kit, as this is the sampling frame from which participants are selected, based on their age [10]. Those who are not enrolled in Medicare are likely to be individuals from poorer socioeconomic backgrounds, with lower English proficiency and living in remote locations, so that the program unintentionally excludes those from more disadvantaged backgrounds, exacerbating the already widening disparities in health that exist. Indigenous people are less likely to be enrolled with Medicare than non-Indigenous people, with estimates from 1997 indicating enrolment rates in the range of 65-80% for Indigenous Australians [92]. It is believed that enrolments have improved over the past decade although recent estimates are not available. Moreover, if addresses recorded by Medicare are not updated - as may be the cases for many transient and mobile Indigenous people - it is likely many people will miss out on receiving a kit.

##### ii) Postal route of FOBT screening kit distribution

Delivery of the FOBT screening kit by post significantly impedes participation, for those who do not have a fixed address (or post box). This particularly affects mobile/transient populations and those living in remote areas or who are homeless, situations more commonly experienced by Indigenous than non-Indigenous Australians. The NBCSP pilot evaluation identified this limitation to participation and receipt of test results [9]. Arguably, options are needed that enable those without fixed address to receive a kit by alternative means, thereby giving them similar access. Pilot activities are taking place in some states in Australia exploring alternative mechanisms of delivering kits to Indigenous people [93]. These include distributing kits from Aboriginal Medical Services, opportunistic distribution of kits, and the utilisation of Aboriginal Health Workers to deliver kits and follow up individuals with kits to encourage them to complete the tests. These innovations are being evaluated.

Despite greater flexibility in distribution being needed, there is no national initiative to improve access for Indigenous people to the NBCSP through alternative targeting strategies. A mechanism is needed whereby those who are interested in receiving a kit are able to obtain one from their health centre, as occurs in Canada where all people over the age of 50 are able to obtain a free FOBT kit from their health care provider [94]. In Australia, the possibility exists of broadly promoting cancer screening through mobile vans visiting Indigenous communities every two years, as is done for breast and cervical screening. This may require addressing logistical difficulties, including the need for the van to stay extra days to give enough time for participants to provide two samples.

##### iii) Role of the General Practitioner

In Australia's program, general practitioners (GP) have a pivotal role to play. They receive FOBT results of patients who nominate them as their GP, manage patients with a positive test and organise referral for colonoscopy. GPs are also responsible for notifying the central registry of patient outcomes and any referrals for colonoscopies [10,95].

GPs have an important role in encouraging test completion [96], however, their pivotal position in the program poses several problems which may limit access to the program as well as to follow-up tests. Indigenous people in general have lower levels of utilization and access to GPs, especially regular GPs. This is particularly true in rural and remote regions [91]. In the case where an individual has received the kit and completed the test but has not nominated a GP on the form, it is up to the patient to initiate follow-up of their test results with a doctor or nurse. A problem arises when an individual has difficulty reading and understanding the letter notifying a positive result and then acting upon it appropriately. This is also a barrier for others like those of CALD background, the elderly and those with disabilities. Additionally, many Indigenous people have poorer continuity of care, given the high doctor turnover in many rural areas and in Aboriginal Medical Services (AMSs). This has led to a large reliance on overseas trained doctors [97] who are often referred to by their first name, thus posing difficulties when identifying a participant's nominated GP. Alternative approaches the program could employ include shifting some responsibility from the doctors to the nurses and health workers within clinics and hospitals. The bowel cancer screening test could also be integrated into health services so when a person of the target age presents with another problem, a screening test is administered by a health worker. At present there are no supplies of screening kits available to AMSs or hospitals. Alternative approaches could also be considered, such as in the UK where screening hubs have been established and are responsible for call and recall of the screening population, issuing and analysing kits, arranging colonoscopies and notifying GPs of patient outcomes [98].

##### iv) Target age group

Despite overall incidence and mortality rates being higher in non-Indigenous people, Indigenous Australians die from CRC at a younger age, before the targeted screening age [10]. For example, in the 45-49 age group mortality rates in Indigenous Australians was 11.3 per 100 000 compared with 7.8 for non-Indigenous people. In the 50-54 year age group the difference is even more marked with a rate of 24.1 for Indigenous people while in non-Indigenous it is 7.8 [10]. Considering the time taken for a polyp to develop into cancer may be over 10 years [38], it is crucial that Indigenous Australians are targeted for screening at a younger age than the recommended 50 years. Reasons for an earlier age of onset of CRC are not clear and need to be identified, but may relate to exposure to higher levels of risk factors at younger ages. In 2005/2006 it was announced that Indigenous people could participate in the NBCSP from the age of 45 [41], but it is not clear whether FOBT kits are in fact being sent out to Indigenous people at this earlier age. Given that this would require Indigenous status to be reliably identified on Medicare enrolment records, a large proportion of at-risk Indigenous people are likely to be missing out on getting screened earlier.

##### v) Health information systems around recording Indigenous status

For a participant to be identified as Indigenous in the NBCSP, they as well as their GP and colonoscopist must *all *record Indigenous status on the appropriate forms including reports that are sent to the central registry. Information on Indigenous status recorded within Medicare is currently voluntary, and a large proportion of Indigenous people are not identified. The lack of recording of Indigenous participation in other screening programs has been noted to affect the ability to determine to what extent screening is making an impact on cancer incidence and mortality [26]. Not knowing how many Indigenous people are completing the test and being followed up, impedes assessing how well the screening program is targeting Indigenous people, and hence the development and modification of the program. Thus, adequate identification is crucial for improving the delivery of screening services to the Indigenous population. The inadequate information systems are reflected in the high loss to follow-up (45%) observed in the NBCSP, both concerning and ethically unacceptable as many people with a positive test could miss out on colonoscopy and potentially lifesaving treatment [25]. Although not a direct barrier, recording of Indigenous status is important in evaluating participation, and targeting younger Indigenous people for a screening kit.

##### vi) Other logistical issues- privacy, storage and test viability

Other logistical barriers relate to an individual's access to privacy needed to do the test and an appropriate place to store samples until a second sample is taken. The way the test is designed at present assumes access to conventional housing with a private toilet. Although the test does not need to be kept refrigerated it is recommended that the sample is kept in a dark and cool place. Many Indigenous people live in substandard housing, sadly, many without functioning facilities. Several families often live within one house, which may compromise the privacy required for test completion.

High temperatures and delays in completing the test are known to affect the sensitivity of the immunochemical FOBT test [99]. This problem has been noted in several European countries and is likely to arise in Australia where many regions experience extremely hot conditions. In remote areas where postal collections are infrequent, many completed kits could remain in post boxes for several days before being collected and delivered to the laboratory.

##### vii) Literacy requirement

Literacy is essentially a pre-requisite for participation and completion of screening tests distributed by post. The initial contact with potential participants is through a written letter and the information material and the screening kit is with limited and limited graphical instructions. An investigation into the impact of health literacy on participation in CRC screening revealed that lower literacy was associated with reduced confidence and perceived self-efficacy in carrying out the test and lower likelihood of completion [100]. Being able to read the instructions is imperative to completing the test correctly; the task and instructions are complicated, relying on a wordy instruction sheet. While the kit provides instructions in a number of languages, Indigenous languages are not included. Potential participants may feel unable or unsure about whether they would be completing it correctly. Although doing the test at home might appear be more appealing and less shameful, correct execution remains a challenge. The requirement to fill identification details on small labels to be stuck on the sample sticks presents another potential obstacle. These literacy-related issues are a major drawback of the postal distribution of test kits where language and poor literacy is a critical, often unaddressed, factor influencing cancer screening behaviour.

The qualitative evaluation report of the NBCSP noted that language and literacy issues were a major barrier for Indigenous Australians with many unable to understand or read the instructions and putting the kit aside after opening it. Furthermore, it was noted that Indigenous people were unlikely to seek assistance if they experienced difficulties. An analysis of those who did not complete the screening test revealed that they were less confident about their ability to take the test and the accuracy of the results. They also had lower levels of English competency, felt overwhelmed with the contents of the kit and confused regarding what had to be sent back. Community health workers were an important source of information and support for Indigenous people compared to GPs and helped to encourage participation. In some cases Indigenous people did not respond until they were contacted by a health worker and many were more interested in undertaking the test once they had spoken to a health worker about it [82]. With test kits mailed to home, an individual's decision about completing the test (or not) is made without discussion with a health practitioner over the benefits and harms of participation. Encouragement and assistance from health personnel is important for facilitating participation.

It is well known that Indigenous people respond to information more effectively through oral and pictorial, rather than written methods of communication [101]; this should be taken into consideration in the future design of the NBCSP. An educational bowel cancer screening flipchart for Indigenous Australians has been developed using pictures to demonstrate how to do the test. Animated video is currently being used in the USA to encourage screening uptake among American Indians [102]. Similar methods could be used in Australia in contexts where such technology is accessible.

##### viii) Nature of the screening test

This issue is underexplored in regard to CRC screening. Although discomfort and uneasiness with handling faecal material could be experienced by almost all participants, little is known about any taboos in Indigenous culture in regard to this. The qualitative evaluation of the pilot did not find any significant issues with this, although it was noted that those selected to participate in interviews were those who were comfortable to discuss the topic [82]. This potentially significant barrier to participation needs further exploration so that alternative approaches could be considered. For example, an individual could provide a stool sample to a clinic where a nurse or health worker could be responsible for completing the test with the kit. This would also ensure the test is completed correctly. Currently Australia only offers one option for CRC screening - the FOBT. Exploring preferences for screening methodologies among different groups and incorporating their preferences into practice is a possibility for increasing adherence to CRC screening [103,104].

##### ix) Barriers to compliance with follow-up and treatment

Completion of the screening test is only one part of the screening process. A positive test must be followed up with appropriate diagnostic evaluation such as a colonoscopy. This step in the screening pathway poses a great challenge for Indigenous people, particularly those living in rural areas, where colonoscopies are not available locally requiring them to travel long distances. Leaving the community and family is known to be a significant barrier to compliance with cancer treatment in Indigenous people [3,74]. Results from the 2009 NBCSP monitoring report show that only 43% of total participants with a positive FOBT were followed up by GPs [10] with inadequate follow-up following a positive FOBT test an identified issue for CRC screening [25,105].

Although the NBCSP monitoring reports indicate no differences between the follow-up of Indigenous and non-Indigenous people, it is likely to be worse among Indigenous and minority populations particularly those living remotely. This area needs to be addressed through intensive follow-up and interventions to encourage confirmation of diagnosis and subsequent access to and compliance with treatment.

#### 2.3 Strategies used for improving participation in CRC screening of Indigenous and ethnic minorities internationally

In recent years there has been a proliferation of literature investigating CRC screening in minority and underserved groups, identifying barriers to screening and examining how program design can be improved to reduce disparities in cancer outcomes. Intervention studies for improving CRC screening uptake and adherence have been undertaken in the USA. However, the number of studies targeting Indigenous people is minimal with the majority focusing on other ethnic minority and low literacy groups including African American, Latino and Chinese immigrants. However, these studies provide some information on what may or may not work in considering implementation in Australia.

The approaches taken have included education and educational sessions, community based interventions, the use of lay health workers, storytelling, mailed interventions, mail-out reminder systems, telephone interviews, and more recently the utilisation of patient navigators and electronic/educational decision aids [106-110]. The majority are tailored to the characteristics of the target group, incorporating their own languages and motifs. A review by Vernon [111] found that those incorporating intensive and personal follow-up appeared to increase adherence more successfully [111]. A more recent systematic review of interventions identified organisational level changes as being most effective at enhancing screening behaviour. Changes included establishing separate clinics for screening and incorporating non-physicians into the screening process [106,112]. Table 5 summarises these intervention studies. The range of interventions trialed highlights difficulties in making comparisons because of differences in outcome measures, target populations, and the type of screening test used. As many of the interventions were carried out in the USA, outcome measures were usually uptake of any type of CRC screening. Interventions to ensure that those with a positive FOBT follow through with colonoscopy are lacking. Patient navigators could play a critical role, helping individuals to overcome many of these barriers in obtaining quality cancer care [113]. Initial evaluation of a small Indigenous women's cancer support group established in regional Western Australia that helped to encourage screening, support families of those with cancer and help patients through their cancer journey could potentially be an effective model for facilitating Indigenous participation in bowel cancer screening while also providing assistance with follow-up tests and treatment [114]. Such community-based and community-led groups could make a significant contribution and further evaluation of their influence on cancer screening behaviours would provide the evidence needed for this.

**Table 5:** Interventions for improving uptake of CRC screening in disadvantaged and minority groups

Approach	Study Type	Screening type	Population group targeted	Observed changes in screening uptake or intent to screen for CRC
***1. Organizational/system changes***				

**Patient navigators to overcome patient reported barriers from screening to treatment **[123]	RCT	Any CRC screening	Low income and non-English speaking	Uptake of CRC screening post-intervention was 27% vs 12% (p‹ 0.001) before intervention.
**Culturally sensitive patient navigators and physician recommendation **[124]	RCT	Endoscopy and FOBT	Low income, Hispanic patients attending primary care practice	Completion of endoscopy 6 months post intervention was 15.8% in intervention group vs 5% in control (physician recommendation only) (p= 0.019) Completion of FOBT 3 months post intervention was 42.1% in intervention group vs 25% in control group (p = 0.086)
**Patient navigator combined with reminder letter **[125]	Intervention	Any CRC screening	English speaking, Immigrants from Brazil, Portugal & Haiti	31% of intervention group vs 9% control patients completed screening after 6 months

***2. Targeting of healthcare users***				

**Culturally appropriate intervention using health educator and bilingual educational material **[126]	RCT	FOBT	Chinese Americans	Uptake of FOBT after 6 months was 69.5% intervention group vs 27% control group

**Personalized and tailored interventions **[127,128] Four groups; 1-Tailored intervention 2-Standard intervention 3-Tailored intervention plus phone reminder 4-Control	RCT [128]	Any CRC screening	Mixture of African American (58%) and white	Screening assessed 2 years post intervention: Screening completion among those in the intervention groups ranged from 44-48% vs 33% control group (p‹0.05) [128]

**Storytelling to promote CRC screening **[110,129]	RCT	Endoscopy	Low SES Latino	Intent to obtain CRC screening via endoscopy increased in those exposed to storytelling compared to those exposed to risk tool based information (p = 0.038)

**Community based awareness and educational interventions **[130]	Participatory using intervention material developed through participatory approach	Any CRC screening	Rural white	Intention to screen increased significantly in those exposed to educational materials and who had not been tested in the last 5 yrs compared to those that had (p = 0.025).

**Community-based participatory **[131] Using culturally relevant approach and education held on a ‘family day'. Followed by intensive follow-up and encouragement of participants by letters and phone calls	Participatory	Any CRC screening	Rural Native Hawaiian in Hawaii	Increase in compliance with CRC screening in both men and women. CRC screening 6 months post intervention increased in men from 39% to 75% (p = 0.002) and in women 36% to 76% (p = 0.002) Limitations- small sample size (28 men and 25 women)

**Culturally appropriate education to enhance knowledge and screening **[132] Assigned to one of three groups: 1. Cultural & self-empowerment group 2. Traditional group 3. Modified cultural group 4. Control	Experimental/repeated measures	FOBT	African-American Elders	Knowledge assessed at 6 and 12 months. FOBT screening assessed at 12 months and found greater participation in those in Cultural & Self Empowerment group.

**Intensive one-on-one patient education **[133]	RCT	FOBT	Ethnically diverse group	Proportion of patients returning FOBT was significantly higher in intensive education group compared with those receiving standard education (65.6% vs 51.3% p‹0.01)

**Telephone support intervention **[134]	RCT	Any CRC screening	Minority, low income women	Proportion of women completing CRC screening increased from 39% to 54% in the intervention group and 39% to 50% in the control group (p = 0.13)
**Tailored telephone outreach **[135]	RCT	Any CRC screening	Minority	CRC screening compliance 6 months post intervention was 27% in intervention group vs 6.1% in control group (mailed print material)

**Education with Elderly Educators **[136] Four educational methods: 1. Elderly Educators 2. Elderly Educators plus Adaption for Aging Changes method 3. Adaption for Aging Changes method 4. Traditional method	2-by-2 factorial design	FOBT	Socioeconomically disadvantaged African Americans and White	Participation in FOBT was 93% in those receiving combination of Elderly Educators plus Adaption for Aging Changes method, 63% in those exposed to Elderly Educators only and 43% participation in those receiving Adaptation for Aging changes method only and 56% exposed to traditional method.

**Video-based education **[137]	RCT	FOBT	Majority had less than high school education	69.6% of intervention group returned FOBT vs 54.4% in control group (p = 0.035)

## Discussion and Conclusion

Bowel cancer screening can provide important benefits and reduce mortality from bowel cancer. However, organised nationwide screening programs such as that introduced in Australian may overlook the needs and beliefs of minority groups, including Indigenous Australians, given their focus on what is most cost effective for the country as a whole. Strategies and interventions at several levels are needed to improve CRC screening uptake and reduce access inequities. These range from policy and program changes to remove many of the structural barriers, to targeting individual, behavioural and attitudinal variables and enhancing provider knowledge and incentives.

There is a need to understand the reasons behind the low participation of Indigenous and CALD groups in greater depth. Research and consultation with Indigenous populations is needed to elucidate the barriers most impacting on participation and to better understand what will work for Indigenous people and other minority groups in Australia - to date, largely unexplored. Results from pilot studies in Queensland and Victoria investigating alternative mechanisms for delivering the screening kits to Indigenous populations will be informative when available, and should shed some light on effective strategies to improve participation. However, as it is unlikely that the screening test of choice will be changed in the near future, several modifications to the current program can be made in the shorter term to enhance access to the program for Indigenous Australians and are detailed in Table 6.

**Table 6:** Recommendations for alterations to Australia's NBCSP to improve access and participation for Indigenous Australians

**Recommendations**
**1) Alternative means of distribution of FOBT test kits**

Provide an alternative mechanism of delivery and return of kit (to target those who may not have a post box or Medicare enrolment). This could entail supplying hospitals and AMSs with kits to distribute to increase opportunistic screening.
**2) Dedicated health personnel for follow-up and support**

Ensure there is a dedicated health worker knowledgeable about the program to follow-up individuals receiving a kit and to provide personalised advice, education and assistance with completing the test. Intensive support of Aboriginal people will be necessary for increasing screening uptake.
**3) Integration of screening into primary care/general chronic disease management**

GPs have an important role in actively encouraging participation in screening [96], however many GPs are neither supportive of or knowledgeable enough about the program nor do they have time to discuss screening with patients [138,139]. Their key role in the program means that whether a patient has a regular GP or not can affect participation and attendance to follow up procedures. (If an individual receives a positive test but has not nominated a GP, then is it is up to the individual to follow up their test result)
The key role of the GP in activating post screening diagnostic and follow up requires reconsideration of alternative approaches using either dedicated health professionals or centralised screening centres to support a greater number of people having access to the screening kit and opportunities for referral. Opportunistic screening through file tagging is also another potential way in which to improve participation [140].
Completion of forms by GPs and colonoscopists should be mandatory as should identification of Indigenous status in order to attain greater quality data that will give an indication of the burden of bowel cancer and how the program impacts on incidence and mortality, and levels of follow-up and treatment. This is also important for the general population. Perhaps greater incentives for health professionals may be needed for this as presently GPs receive $7.70 for each form submitted [95].
The administrative role of GPs in the program is poorly defined and needs attention with accountability and responsibilities clarified, and appropriate interventions implemented to increase GP's awareness of their roles and responsibilities [95].
**4) Improve health promotion and availability of culturally relevant educational materials**

Greater coverage of bowel cancer screening in health promotion campaigns and the media is needed to increase general knowledge and awareness in the population. This will also help to remove the shame and stigma associated with discussing bowel cancer. Promotional activities should occur prior to individuals receiving the kit so that there is some awareness and expectation of the test.
Increase the availability of culturally appropriate, Indigenous-specific educational resources, if possible in local languages and including local terms for main parts of the body. Translated materials were available in 13 languages for CALD groups during the pilot therefore it should be possible to make information and brochures available in Indigenous languages.
To overcome the literacy barrier, greater emphasis on pictorial methods of education including videos and diagrams should be included with the screening kit.
**5) More community-based participatory research into Indigenous understandings and perceptions of bowel cancer**

Further research into Aboriginal understandings and perceptions of CRC and CRC screening, including knowledge, beliefs and attitudes is necessary to inform appropriate approaches for intervention and resources. This includes a greater emphasis on participatory methods of health promotion.
**6) Implement ongoing annual or biennial screening for CRC**

Currently the NBCSP is offering only once of testing to the Australian population and only to those in the specified age brackets. Provision of funding for ongoing rounds of screening are necessary for not only targeting a greater number of people and enhancing opportunities for screening uptake, but allowing time for familiarization with the program[141].
**7) Ensure Indigenous Australians have access to FOBT kits from the age of 45 years**

Given the younger age at which CRC is occurring among Indigenous people, consideration should be given to ensuring screening kits are available to Indigenous Australians from the age of 40-45 years.

The nature of the NBCSP may exacerbate the wide disparities in cancer outcomes that exist between Indigenous and non-Indigenous Australians; the programme design favours participation by the majority and unintentionally excludes disadvantaged and remote populations, perpetuating prevailing health inequities. This review provides evidence that the program needs to be modified to facilitate access to and participation by Indigenous people and other minority populations through a more community-based, people-friendly approach, better integrated into primary health care. Modifications such as those we have described are needed, taking into account epidemiological differences, life circumstances, and specific participation barriers experienced by Indigenous people.

## Competing interests

The authors declare that they have no competing interests.

## Authors' contributions

AC undertook the literature search and other sourcing of relevant information and the initial draft of the paper. JK assisted with refinement, shaping and editing of the manuscript. ST was involved in the initial conception of the work, sourcing funding and in refinement and editing of the manuscript. All authors approved the final manuscript.

## Pre-publication history

The pre-publication history for this paper can be accessed here:

http://www.biomedcentral.com/1471-2458/10/373/prepub
